# PixCell: A generative foundation model for digital histopathology images

**Published:** 2025-06-05

**Authors:** Srikar Yellapragada, Alexandros Graikos, Zilinghan Li, Kostas Triaridis, Varun Belagali, Saarthak Kapse, Tarak Nath Nandi, Ravi K Madduri, Prateek Prasanna, Tahsin Kurc, Rajarsi R. Gupta, Joel Saltz, Dimitris Samaras

**Affiliations:** 1 Stony Brook University; 2 Argonne National Laboratory; 3 The University of Chicago

## Abstract

The digitization of histology slides has revolutionized pathology, providing massive datasets for cancer diagnosis and research. Contrastive self-supervised and vision-language models have been shown to effectively mine large pathology datasets to learn discriminative representations. On the other hand, generative models, capable of synthesizing realistic and diverse images, present a compelling solution to address unique problems in pathology that involve synthesizing images; overcoming annotated data scarcity, enabling privacy-preserving data sharing, and performing inherently generative tasks, such as virtual staining. We introduce **PixCell**, the first diffusion-based generative foundation model for histopathology. We train PixCell on PanCan-30M, a vast, diverse dataset derived from 69,184 H&E-stained whole slide images covering various cancer types. We employ a progressive training strategy and a self-supervision-based conditioning that allows us to scale up training without any annotated data. PixCell generates diverse and high-quality images across multiple cancer types, which we find can be used in place of real data to train a self-supervised discriminative model. Synthetic images shared between institutions are subject to fewer regulatory barriers than would be the case with real clinical images. Furthermore, we showcase the ability to precisely control image generation using a small set of annotated images, which can be used for both data augmentation and educational purposes. Testing on a cell segmentation task, a mask-guided PixCell enables targeted data augmentation, improving downstream performance. Finally, we demonstrate PixCell’s ability to use H&E structural staining to infer results from molecular marker studies; we use this capability to infer IHC staining from H&E images. Our trained models are publicly released to accelerate research in computational pathology.

## 1 Introduction

Generative models, capable of controlled synthesis of realistic tissue images, present a compelling solution to scarcity of rare disease presentations, to annotated data scarcity, to the need for privacy-preserving data sharing, as well as to carry out inherently generative tasks, such as virtual staining. The rise of computational pathology has brought significant advances in automating clinical tasks, such as tumor subtyping and tumor classification. However, developing robust models in this field is often constrained by data-related challenges. For instance, accurate tumor-infiltrating lymphocyte segmentation models, essential in making some clinical predictions, require extensive pixel-level annotations, which are laborious and costly to acquire. Thus, the prevailing approach has been to train powerful self-supervised learning (SSL) models, which benefit from vast amounts of unlabeled data and can be readily adapted to perform downstream tasks.

Self-supervised learning (SSL) models (or vision-language models) are trained to discriminate between different images [13, 71, 19, 50] (or image-text pairs [28, 29, 47, 73]), a process that entails learning to extract discriminative representations from a given image. These representations are then utilized to solve a given downstream task, and have been shown to indeed encode useful information for making predictions [13, 19, 47].

Although SSL provides a clear pathway for solving most computational pathology tasks, there are some fundamental drawbacks. Firstly, using a pre-trained self-supervised model to solve any downstream task involves training a prediction ‘head’, which requires a non-trivial amount of high-quality annotated data. Secondly, SSL training benefits from curating diverse datasets to train on Regulatory, data privacy and data ownership issues frequently limit data sharing, therefore, in most cases each institution or consortium ends up training their own SSL model primarily with private data supplemented by a few commonly used public datasets such as the TCGA whole slide imaging dataset [72]. Data sharing challenges limit model performance and can potentially create bias due to the specific demographics of training datasets [69, 35]. Finally, tasks where we synthesize a completely new image from a given slide, such as virtual staining (stain translation) [7, 54, 40], are difficult to perform with models exclusively trained to discern between different images.

We propose to overcome these limitations using a generative model. We introduce **PixCell**, the first diffusion-based generative foundation model for histopathology. We employ a Diffusion Transformer [55], and train PixCell on **PanCan-30M** – an extensive dataset of 30.8 million 1024 × 1024 px image patches derived from 69,184 H&E-stained whole slide images (WSIs), covering a comprehensive range of cancer types. To effectively scale up training we employ: (a) progressive training, starting from 256 × 256 patches and gradually increasing the generated image size to 1024 × 1024 and (b) per-patch guidance, by conditioning PixCell on feature embeddings from a pre-trained self-supervised model (UNI-2h [13]), providing rich image semantics to guide the generation process.

PixCell achieves the state-of-the-art generation quality in 256 × 256 and 1024 × 1024 image generation. Benefiting from the pan-cancer training, PixCell significantly outperforms prior models trained on single cancer types [21, 77, 38]. To highlight the utility of PixCell-generated data, we train an SSL encoder exclusively on synthetic samples. This encoder achieves comparable performance to a model trained on corresponding real images, which underscores that PixCell’s synthetic data can serve as an effective, privacy-preserving replacement for real data in training downstream models. We release this synthetic dataset and invite the community to experiment with the synthetic images.

PixCell also facilitates fine-grained controllable generation. Using a ControlNet [78], we synthesize realistic histopathology images conditioned on cell segmentation masks and demonstrate how these synthetic image-mask pairs can augment the training dataset and improve the performance of a downstream segmentation model. Furthermore, PixCell demonstrates generalization capabilities, successfully synthesizing images with staining techniques unseen during training. We investigate PixCell’s adaptability to stain translation, by fine-tuning the model on a dataset composed of approximately paired H&E and IHC images. PixCell achieves SoTA performance in stain translation, showcasing its potential as a versatile foundation model that can be efficiently adapted to specialized generative tasks using limited additional data.

In summary, our contributions are as follows:

1. We develop **PixCell**, the first generative foundation model for histopathology images.
2. We demonstrate state-of-the-art image generation quality, producing high-fidelity and diverse samples that can be used in place of real images for training self-supervised models.
3. We explore PixCell’s capability for controllable image generation by conditioning on cell segmentation maps, and showcase its utility through synthetic data augmentation.
4. We illustrate PixCell’s ability to generalize to different staining techniques, which we use to translate from H&E-stained images to IHC, achieving state-of-the-art results.
5. We publicly release the PixCell model weights and associated code to foster further research in computational pathology.

## 2 Method and Data

### 2.1 Training dataset: PanCan-30M

**Figure 1:**
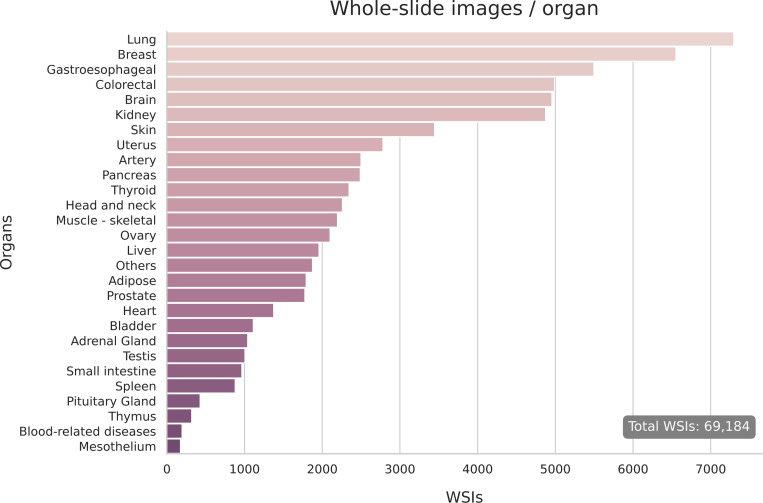
Distribution of organs in the whole-slide images used for the PanCan-30M dataset.

Our training cohort comprises 69,184 Whole Slide Images (WSIs), aggregated to capture a wide variety of tissue morphologies and cancer subtypes. Inspired by Phikon-v2 [19], we source the WSIs from major public repositories including The Cancer Genome Atlas (TCGA) [72], the Clinical Proteomic Tumor Analysis Consortium (CPTAC) [17], the Genotype-Tissue Expression (GTEx) project [44], and other public sources [14, 66], alongside an internal cohort (SBU). The pan-cancer nature of this WSI collection, covering a wide variety of organs, provides a diverse cohort for training a foundation generative model. In Figure 1 we provide a breakdown of the WSIs per organ type used in training.

From these WSIs, we extract 30 million (30,819,977) patches, creating **PanCan-30M**. Each patch is 1024 × 1024 pixels in size, and is obtained from slide regions corresponding to 20 × objective magnification (0.5 microns per pixel). We use the code from DS-MIL [39] for both patch extraction and tissue thresholding. The slides included in the dataset are stained with Hematoxylin and Eosin (H&E), and we do not use stain normalization. See Table 1 for an exact breakdown of the data sources for the extracted patches. From this data, we create a separate hold-out test set of 2.3 million image patches, which we call **PanCan-Test**. In Appendix Figure 15 and Table 9 we provide an exhaustive breakdown of the dataset per organ and data source.

### 2.2 PixCell diffusion model

PixCell is a Diffusion Transformer (DiT) model [55], with its architecture adapted from the PixArt-σ framework [10]. We follow the LDM approach [58], operating in the latent space of the pre-trained Variational Autoencoder (VAE) from Stable Diffusion 3 [18]. The VAE first encodes the high-resolution histopathology images from PanCan-30M into compressed latent representations, on which we train the diffusion model. The VAE encoder has a downsampling factor of × 8, compressing a 1024 × 1024 × 3 image to a 128 × 128 × 16 latent. We opted for the Stable Diffusion 3 VAE, despite the larger latent representation (16-dim in SD3 vs 4-dim in SDXL [56]), because of the better reconstruction quality in pathology images (average PSNR of 31.79 for SD3 vs 26.93 for SDXL ^1^).

To guide PixCell during generation, we condition the model on image embeddings extracted using the self-supervised UNI-2h [13] encoder. In natural images, generative foundation models are usually trained on extensive image and text caption pair datasets. For digital pathology images, as there is no large-scale image-caption dataset that we could use to train a generative model on, we can utilize SSL embeddings in place of the text captions [21]. By conditioning on SSL embeddings, we replace the text prompt given in a text-to-image inference with a reference image ‘prompt’. The embeddings are integrated into the DiT blocks via cross-attention.

#### 2.2.1 Progressive training

Instead of directly training PixCell on the 30M 1024 × 1024 image patches, we employ a progressive, multi-stage training strategy with increasing image resolution to optimize efficiency and model performance. This technique is also used for the PixArt family of models [12, 10] in the natural image domain.

We split training into three stages that utilize the same underlying PanCan-30M dataset of 1024 × 1024 pixel patches. For the lower-resolution training stages, multiple smaller patches are derived from each original high-resolution patch. To save compute time, we pre-extract VAE features for all 1024 × 1024 patches in our PanCan-30M dataset, using the pretrained SD-3 VAE [18], resulting in 128 × 128 × 16 dimensional latents. Simultaneously, we extract the 16 × 1536 dimensional conditioning embedding using the pretrained UNI-2h [13] encoder, corresponding to one embedding for each 256 × 256 patch in the image.

We also create a separate hold-out test set of 2.3 million image patches, which we call **PanCan-Test**. We utilize the pre-extracted VAE features and UNI-2h embeddings corresponding to the remaining 27.9 million image patches for all training stages of PixCell:

##### Stage 1 - Low-resolution training (PixCell-256):

**Figure 2:**
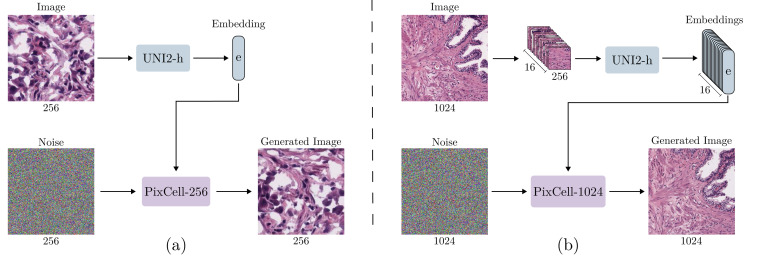
Overview of (a) the PixCell-256 model and (b) the PixCell-1024 model. Each model is conditioned on the UNI-2h embeddings that describe the given image.

We first train a base model on latents corresponding to 256 × 256 px images. For each 1024 × 1024 image’s pre-extracted features, we generate 16 non-overlapping 32 × 32 × 16 crops from its 128 × 128 × 16 VAE latent. Each of these latent crops is then paired with the corresponding 1 × 1536 embedding from the original 16 × 1536 UNI-2h embeddings. This stage is trained for one epoch over the entire dataset, yielding the **PixCell-256** model (Figure 2 (a)).

##### Stage 2 - Intermediate fine-tuning:

The weights from PixCell-256 are used to initialize the model for a second stage of fine-tuning, this time using 512 × 512 images. From each full-resolution VAE latent, we extract 4 non-overlapping 64 × 64 × 16 crops. Each 512 × 512 equivalent latent crop is paired with its corresponding 4 × 1536 embeddings (representing a 2 × 2 block of tokens) from the original UNI-2h embeddings extracted. This stage serves to gradually adapt the model before the final high-resolution training.

##### Stage 3 - High-resolution fine-tuning (PixCell-1024):

Finally, the model from the 512 × 512 stage is further fine-tuned on latent representations corresponding to full 1024 × 1024 pixel images for one epoch. The final 1024 × 1024 training stage directly uses the full pre-extracted 128 × 128 × 16 VAE features and the complete 16 × 1536 UNI-2h embeddings. This final stage produces the high-resolution **PixCell-1024** model (Figure 2 (b)).

Table 2 summarizes key training parameters, such as the number of iterations and batch sizes, for each stage. Across all stages, we use the AdamW optimizer with a weight decay of 0.03 and a constant learning rate of 2 × 10^−5^. We trained our models on a cluster with several NVIDIA A100 GPUs. More details are given in Appendix A.2.

### 2.3 Controllable generation

PixCell generates images conditioned on an embedding from a self-supervised encoder. Within this framework, we can synthesize variations of a given image but lack further control over the synthesized image content. To introduce the ability to controllably generate images, we employ ControlNets [78], which add image-level control to pre-trained diffusion models.

**Figure 3:**
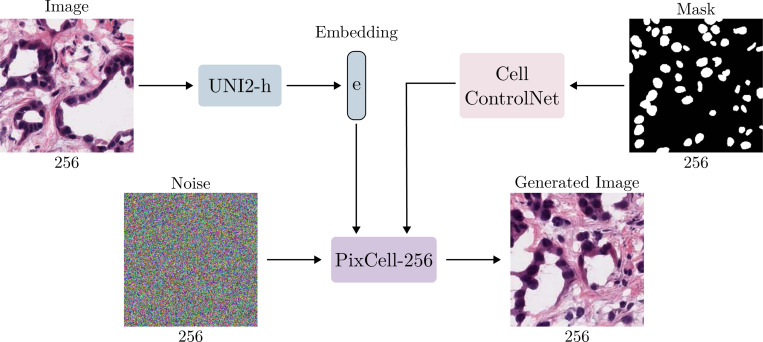
PixCell-256 with a cell mask ControlNet. The two sources of conditioning allow for fire control over the generated image: the UNI embedding dictates the style of the generated image while the cell mask guides the cell layout.

We train a ControlNet for PixCell-256 to guide generation with a cell layout mask. To construct a training dataset, we utilize a pre-trained CellViT-SAM-H [26] model trained on 0.5 microns per pixel images. We extract cell masks from 10,000 images of all cancer types from PanCan-30M and train the ControlNet with image, UNI embedding, and mask triplets. The diffusion transformer ControlNet copies each layer of the base transformer, adding an intermediate output linear layer that is zero-initialized to combine the base transformer and ControlNet transformer features [11]. An overview of the ControlNet approach is provided in Figure 3.

### 2.4 Stain Inference

PixCell is trained to generate H&E-stained histopathology images conditioned on UNI-2h embeddings. However, the UNI encoder has been trained on both H&E and immunohistochemistry (IHC) images. Therefore, we examine whether our model can generalize to produce images with different stains using different conditions.

To test this, we prompt PixCell-256 using an IHC image from the MIST-HER2 dataset [40]. We extract the UNI-2h embedding of the IHC image and use it to condition the PixCell model. The generated images (Appendix Figure 11) follow the appearance and semantics of the reference IHC patch, showing that, although unseen during training, the model can infer results that would have been obtained with different stains.

IHC stains reveal the spatial distribution of specific molecular markers and are usually significantly more expensive to obtain than H&E. Given that we can generate IHC images, we propose to utilize the pre-trained PixCell model to perform stain translation between H&E and IHC images. Given a dataset of ‘roughly’ paired H&E and IHC images, such as MIST [40], we introduce a method to generate an IHC-stained image from a given H&E image of the same tissue region. Using a generative model for virtual (re-)staining has the potential to enable further downstream analysis of H&E-stained tissues, without requiring an expensive and likely unavailable IHC staining procedure.

We first extract the UNI embeddings of the paired patches for the H&E and IHC images. We then train a rectified flow model [43] that learns to transform UNI embeddings of H&E images to UNI embeddings of IHC images. We chose a rectified flow instead of a diffusion model because of its direct applicability to learning transformations between two data distributions. Then, for a new H&E image, we first extract the UNI embeddings of each patch, transform them to IHC embeddings, and generate the corresponding IHC image. A main advantage of this conditioning-transformation approach is that we operate entirely on a patch level, which is desirable as most of the paired H&E and IHC datasets lack pixel-perfect alignment.

**Figure 4:**
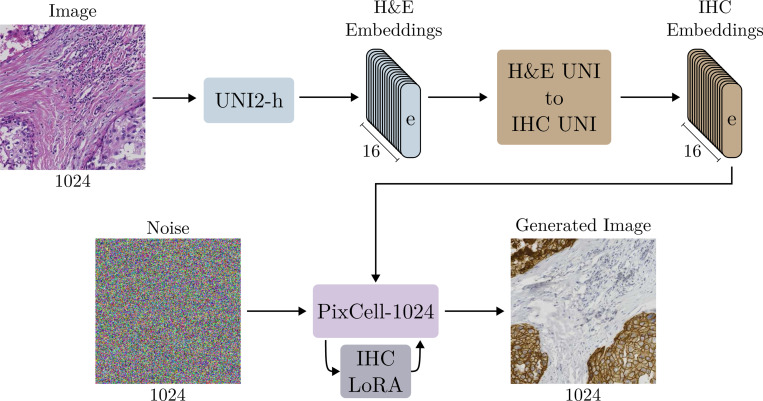
Overview of our approach for translating H&E stained images to IHC using PixCell-1024. We train an additional H&E-to-IHC model that transforms the UNI conditions from one domain to the other. Adding a low-rank adapter to the PixCell-1024 weights significantly improves the synthesized image quality with minimal additional costs.

To further boost the visual quality of the generated IHC data, we show that we can also train a lightweight low-rank adaptation (LoRA) module [27] on top of the PixCell-1024 model, tailored to generating images from the target dataset. Training a LoRA adapter is cheap and only requires the unpaired IHC images. We provide an overview of the proposed stain translation approach in Figure 4.

## 3 Experiments

### 3.1 Image quality evaluation

We first describe the metrics used to evaluate the quality of images generated by PixCell at various resolutions. For 256 × 256 patches, we compute the following metrics:

#### Vanilla FID:

We measure the Frechet Inception Distance (FID) [23] between PixCell-generated and real images, using the implementation of Clean-FID [53].

#### Embedding Similarity:

We measure the similarity of real and corresponding synthetic images in the embedding space of several pretrained image encoders by calculating the cosine similarity between their respective embeddings. For a robust evaluation, we use UNI-v1 [13], Phicon-v2 [19], and CONCH-v1 [47] as the feature extractors.

For 1024 × 1024 high-resolution images, we adopt an evaluation strategy consistent with LRDM [21] and MultiDiffusion [5]. This strategy involves two primary perceptual metrics:

#### Crop FID:

We compare the distribution of 256 × 256 random crops we extract from the synthesized images against the distribution of real 256 × 256 patches using the vanilla Inception-v3 network [65].

#### CLIP FID:

We measure the FID directly between the synthesized images generated by PixCell-1024 and real 1024 × 1024 images. For this, we extract features using a pretrained CLIP ViT-B/32 model [57, 36], which is better-suited to evaluating larger images (> 224 × 224 px).

We sample 10,000 images to measure Vanilla FID and Embedding similarity, following [21, 75]. For large images, we generate 3,000 images for CLIP FID and extract random crops from those images for Crop FID. Unless otherwise specified, we employ the DPM-Solver [45] with 50 sampling steps and utilize classifier-free guidance [25].

#### 3.1.1 Classifier-free guidance scale

To determine the optimal classifier-free guidance scale hyperparameter, denoted as w, we perform a grid search across the guidance values $w\in \left\{1,1.2,1.4,1.6,1.8,2,3,5\right\}$. For this search, we generate images conditioned on randomly selected UNI-2 embeddings from the training set and measure FID against images from the PanCan-Test set. Table 3 indicates that the best image quality (lowest FID) is obtained for w=2 for PixCell-256 and w=1.2 for PixCell-1024.

The optimal guidance scale is noticeably lower than the scales regularly used in text-to-image diffusion models for natural images (typically around 6). We attribute this to our choice of conditioning; whereas a single text caption can characterize different images (wider distribution), a UNI embedding corresponds to a much smaller set of images, i.e., the distribution of images the model learns to sample from given a UNI embedding is more concentrated. We use the optimal guidance values for all image generation tasks reported in this paper, unless stated otherwise.

#### 3.1.2 PixCell-256 results

**Figure 5:**
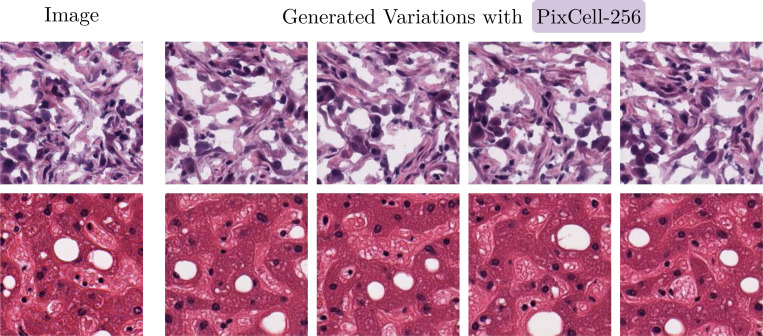
Generating variations of a reference image using PixCell-256.

Figure 5 showcases synthetic samples generated with PixCell-256. We evaluate the image quality of PixCell-256 by comparing its performance against two prior diffusion models: LRDM [21] and ZoomLDM [77]. We conduct our comparison by measuring vanilla FID on images from the PanCan-Test set, as well as the TCGA-BRCA dataset, on which both prior works trained their models.

The results, presented in Table 4, show that PixCell-256 outperforms both prior works on the diverse PanCan-Test set, highlighting the robust generalization capabilities and the benefits of PixCell-256’s pan-cancer training approach. On TCGA-BRCA, PixCell lags slightly behind the other methods, which can be attributed to both being trained exclusively on TCGA-BRCA data.

Both previous models (LRDM [21], ZoomLDM [77]) also use image embeddings from a reference image to generate new images. Therefore, we assess the fidelity and faithfulness to the original image used in generating a patch by measuring the embedding similarity between real and synthetic patches generated from each model on the PanCan-Test set. Table 5 shows that PixCell-256 obtains significantly higher similarity than prior work across multiple pretrained encoders. This further underscores the advantage of our scaled-up pan-cancer training, indicating that PixCell-256 can better preserve essential semantic content from the reference images during synthesis. This ability of PixCell to create new images that retain the properties of a reference image is what we exploit in Section 3.2 to perform self-supervised learning exclusively on synthetic data.

#### 3.1.3 PixCell-1024 results

**Figure 6:**
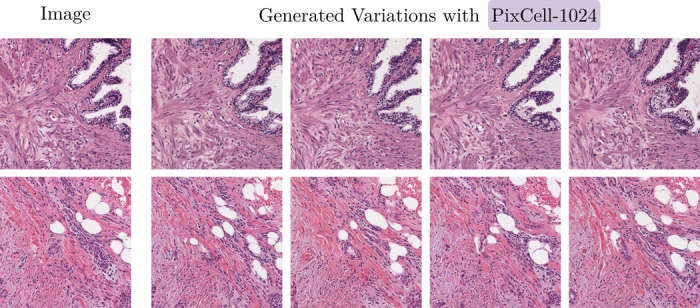
Generating variations of a reference image using PixCell-1024.

We evaluate the performance of PixCell-1024 for high-resolution 1024 × 1024 image generation on the TCGA-BRCA dataset, comparing it against prior methods: LRDM [21], ∞-Brush [38], and ZoomLDM [77]. Our assessment considers image quality, using Crop FID and CLIP FID, as well as generation efficiency, measured in generation time per image (seconds). Figure 6 shows synthetic samples generated with PixCell-1024.

As shown in Table 6, PixCell-1024 demonstrates SoTA performance across all evaluated metrics. For image quality, it achieves a Crop FID of 7.92 and a CLIP FID of 0.68, substantially lower than prior work. This signifies that images generated with PixCell-1024 more closely resemble the feature distribution of real images, compared to those from baseline methods. Furthermore, PixCell-1024 achieves the best generation speed, producing a 1024 × 1024 image in just 2.5 seconds, significantly faster than its counterparts. These findings, coupled with the strong semantic fidelity shown by PixCell-256, highlight the scalability of our PixCell architecture and the effectiveness of the progressive, pan-cancer training strategy from 256 × 256 up to 1024 × 1024 resolution.

### 3.2 Synthetic SSL

In Section 3.1.2, we showed that PixCell-256 synthetic images closely follow the semantics and appearance of the reference image used for extracting the UNI-2h conditioning. This allows us to generate synthetic variants of entire pathology datasets and use them in place of real data. Although this does not guarantee complete data privacy, as generative models have been prone to leaking training samples [8], it is a first step towards enabling synthetic data sharing for model training.

We evaluate the quality of PixCell-generated synthetic images by training an SSL encoder on the synthesized data and comparing its performance against an SSL encoder trained only on real images. To create the real dataset, we sample 1M patches from our internal SBU 12M cohort at 256 × 256 resolution. We name this dataset Real SBU-1M. Next, using PixCell-256, we generate 1M synthetic images by extracting the UNI embedding and generating a new image for each reference image in Real SBU-1M. We call this dataset Synthetic SBU-1M.

We train a ViT-S DINOv2 [52] model for 90 epochs on the Real SBU-1M and Synthetic SBU-1M datasets separately. For evaluation, we use the Thunder benchmark tool [37] to assess the k-NN performance on patch classification. We choose a training-free k-NN evaluation to ensure that the SSL encoder trained on synthetic images is never adapted to real data, making this setting well-suited to measure the effectiveness of the PixCell-generated dataset.

As presented in Table 7, the SSL encoder trained with synthetic images matches the performance of the SSL encoder trained exclusively on real data. The synthetic images can be used as a drop-in replacement of the real images during SSL training without any degradation in downstream performance. We envision that SSL encoders trained on a mix of real and synthetic data will further push the boundaries of self-supervised learning, as they will further scale the training dataset sizes and potentially enable a way to share data between institutions. We release the Synthetic SBU-1M dataset and invite the community to experiment with our synthetic data.

### 3.3 Controllable generation

#### 3.3.1 ControlNet training

To generate 256 × 256 images conditioned on cell segmentation masks, we employed a pre-trained CellViT-SAM-H [26] model trained on 20 × pathology images. We randomly sampled 10,000 images from PanCan-30M and their UNI-2h embeddings, extracted binary cell masks using CellViT, and trained the ControlNet model for 25,000 iterations, using a batch size of 4 and the AdamW optimizer with a learning rate of 1 × 10^−5^.

**Figure 7:**
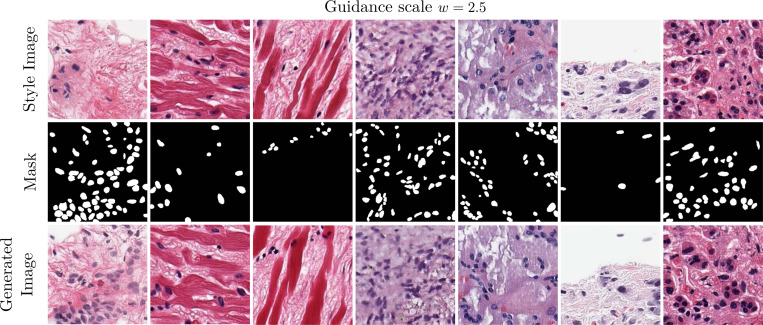
Images generated using the cell mask ControlNet. The synthesized samples follow the appearance of the style image, from which the UNI conditioning is extracted, and the cell layout of the reference mask (columns 1–5). The UNI conditioning from the style image is not sterile from cell information, and when the two significantly contrast, the generated images fail to follow the mask accurately (columns 6–7).

**Figure 8:**
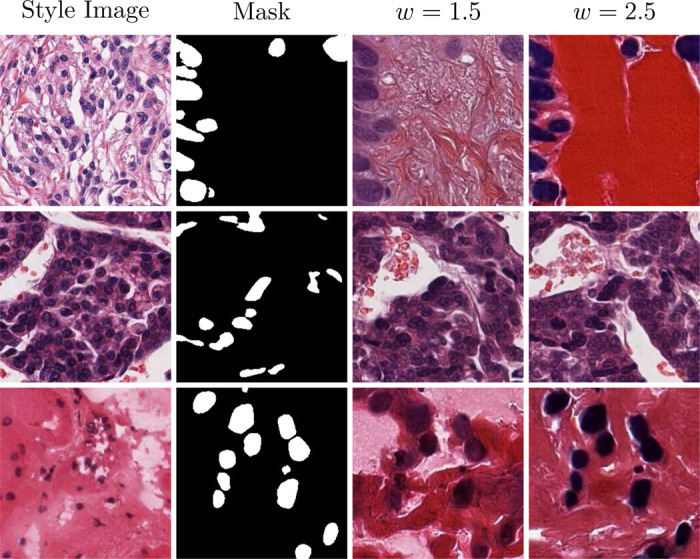
Effect of the guidance scale on the cell mask ControlNet generation.

In Figure 7, we showcase images sampled using the trained cell mask ControlNet. The PixCell-256-Cell-ControlNet model allows for finer control over the generated image. By providing the UNI embedding from a reference image and a target cell mask, we have disentangled the appearance (guided by UNI) and cell layout (guided by the mask) of the generated image. However, this disentanglement is not perfect since the UNI-2h conditioning also encodes some information about the cell count and layout. We observe that in some cases, the clashing control signals lead to images that do not exactly follow the given mask (columns 6–7 of Figure 7). Although we can increase the guidance scale of the ControlNet conditioning, this can lead to potentially unwanted artifacts in the generated images or the model completely ignoring the UNI conditioning, as shown in Figure 8.

#### 3.3.2 Targeted data augmentation

Having trained the cell mask ControlNet, we set up a testbed for data augmentation using synthetic data from our model. We utilize the Lizard dataset [20], which provides images at 20 × magnification alongside their cell segmentation masks. We opt for binary cell segmentation, which our ControlNet is guided by.

As a baseline, we train a segmentation head using features from the last layer of UNI-2h. We use the convolutional segmentation head from the EVA framework [31] that combines the extracted features with the input image to better capture cell edges. The segmentation head is trained on the Lizard data splits from GlaS, PanNuke, and CoNSeP, and is evaluated on the CRAG and DigestPath splits. We specifically selected those splits to exacerbate the difference between the training and evaluation data, testing the generalization limits of the extracted features.

We then utilize the PixCell-256-Cell-ControlNet to perform targeted data augmentation on the evaluation data. For each image in the evaluation set, we extract its UNI-2h embedding and pair it with a random training set mask. We then synthesize a new image using the two sources of conditioning. The generated image follows the appearance of the evaluation set images and has a known ground truth cell mask. We use this synthetic data as additional training samples to re-train the segmentation head. Note that this targeted data augmentation scheme does not require ground truth masks from the test set.

For the evaluation, we measure the accuracy, Dice score, and IoU between the predicted and ground truth evaluation masks. We find that with our PixCell augmentations, the accuracy increases from 0.857 → 0.890, Dice score 0.629 → 0.653, and IoU from 0.751 → 0.802. This improvement validates our observation that by training the cell mask ControlNet we attain finer control over the appearance and cell layout of the generated images, which we can then use to synthesize additional training data for downstream tasks.

#### 3.4 Synthesizing IHC stain patterns using H&E images

We use the MIST dataset [40] to evaluate our stain translation approach. In Appendix Figure 11, we randomly selected patches from the MIST-HER2 slides to extract UNI embeddings from, and show that the base PixCell-256 model can effectively synthesize IHC images without ever being trained explicitly to do so.

To learn the H&E UNI to IHC UNI mapping, we use paired 256 × 256 patches from MIST to train a rectified flow model [43]. Rectified flows learn to transform samples between two distributions, in this case, H&E UNI embeddings and IHC UNI embeddings. To implement the rectified flow model, we use a simple residual MLP network [41] and the roughly aligned H&E and IHC patches from the MIST-HER2 dataset. For each pair of images, we extract the two corresponding UNI embeddings and train the rectified flow model to learn the velocity field from H&E to IHC embeddings. During sampling, we start from an H&E UNI embedding and follow the ODE defined by the trained rectified flow to sample a corresponding IHC embedding. The rectified flow model is trained for approximately 50,000 iterations using a batch size of 32 and the AdamW optimizer with learning rate 1 × 10^−4^.

**Figure 9:**
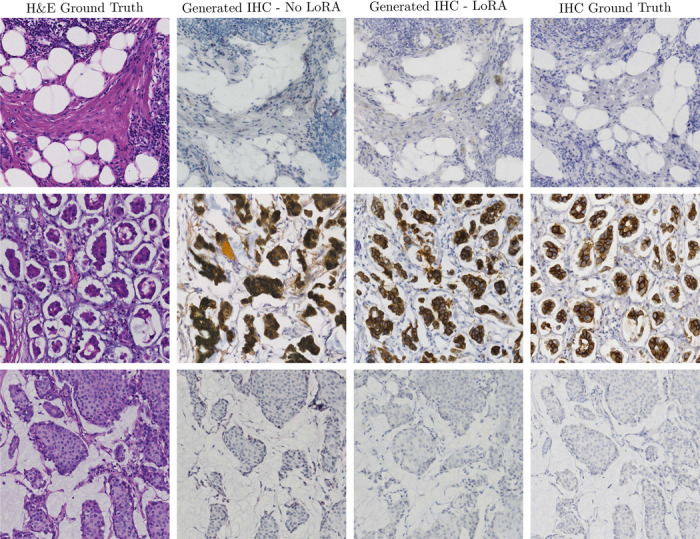
Results for H&E → IHC stain translation using PixCell-1024 with and without LoRA fine-tuning. The quality of the generated images significantly improves with a lightweight low-rank adapter.

In Figure 9 we present the results of this stain translation. We select H&E images from the test set, extract their UNI embeddings, transform them to IHC embeddings with the trained rectified flow model, and generate corresponding IHC images. The resulting samples follow the overall semantics of the ground truth IHC images but noticeably lack in visual quality. Apart from not being trained on IHC data, we attribute the worse quality to the MIST dataset being at a slightly different resolution (0.4661 microns per pixel) than the dataset used for training (0.5 microns per pixel).

To improve the quality of the generated images, we show that we can train a lightweight low-rank adapter [27] for the target dataset. Training a LoRA requires minimal computational resources compared to fine-tuning the entire diffusion model. We train the LoRA on the unpaired MIST-HER2 images for approximately 10,000 iterations, on a single NVIDIA A5000 24GB GPU. The adapter massively improves the generated IHC image quality, without modifying any other component of the stain translation pipeline (Figure 9).

**Figure 10:**
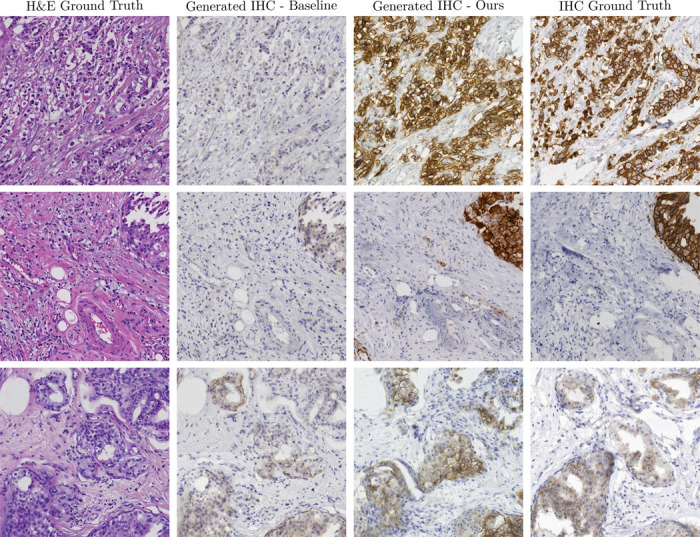
Results for H&E →IHC stain translation using PixCell-1024 and CycleGAN baseline. The baseline fails to translate critical features between the H&E and IHC images.

Finally, we compare the proposed stain translation to the baseline proposed by the MIST paper, which utilizes a specialized CycleGAN trained for translation on the MIST data. In Table 8 and Figure 10 we provide quantitative and qualitative results, respectively. For quantitative evaluation, we measure the SSIM between the generated and real test images, as well as perceptual metrics (FID, KID, Crop FID). Our LoRA stain translation surpasses the capabilities of the baseline model, which fails to capture critical morphological features in the synthesized IHC images, evident in both the perceptual quantitative metrics and qualitative examples provided.

Our foundation-scale pertaining is the key to effectively performing stain translation. Translating between stains requires a broad understanding of the given tissue features, which we posit is impossible to capture by training only on small datasets. The PixCell model can synthesize immunohistochemistry (IHC) stain patterns from routine H&E slides, reproducing spatial biomarker distributions that normally require separate physical assays for each marker. This virtual staining preserves tumor heterogeneity and microenvironment features within the native tissue context, enabling accurate tumor subtyping and treatment selection. The approach reduces the need for labor-intensive serial sectioning and expensive multiplex assays, rapidly producing pixel-aligned, multiplexed IHC images from a single H&E specimen. Methods of this kind can be used in translational research studies, in low-resource settings, in clinical situations where there is limited tissue availability, or potentially in clinical scenarios as a screening method with molecular confirmation.

## 4 Related Work

### 4.1 Self-Supervised Learning in Computational Pathology

The digitization of histology slides into Whole Slide Images (WSIs) has revolutionized pathology, providing a vast amount of visual data for cancer diagnosis and biomedical research. These gigapixel-scale images capture complex tissue architecture and cellular morphology, offering substantial potential for computational analysis. However, the sheer size of WSIs necessitates processing them at the patch level. A significant challenge arises from the lack of granular, patch-level annotations, making traditional supervised learning approaches difficult. This annotation bottleneck has driven the significant traction of foundation models in digital pathology, particularly self-supervised learning (SSL) at the tile-level [13, 71, 19, 50, 1, 74], which allows models to learn meaningful representations from large unlabeled datasets.

SSL enables models to learn generalizable features from the inherent structure of the data itself through pretext tasks. Joint-embedding SSL (JE-SSL) methods, a prominent subclass, aim to learn effective representations by promoting alignment between embeddings of augmented views of the same image while ensuring diversity across the entire dataset. Self-distillation variants within JE-SSL, such as the DINOv2 framework, have demonstrated considerable success in natural image understanding and have been widely adopted in computational pathology (CPath) [9, 79, 52]. These tile-based SSL models, including notable examples like UNI [13], Virchow [71], Prov-GigaPath [74], and Phikon [19], are trained on increasingly large and diverse datasets. Early efforts often utilized public repositories like The Cancer Genome Atlas (TCGA), while more recent models leverage extensive proprietary datasets, encompassing millions of slides and billions of tiles. This data often spans multiple institutions, disease states, tissues of origin, and includes a growing variety of staining techniques beyond hematoxylin and eosin (H&E), such as immunohistochemical (IHC) and special stains, as well as data from multiple image magnifications.

Beyond SSL, other discriminative models leverage contrastive learning with paired image-text datasets [28, 29, 47, 73, 64]. Addressing the gigapixel scale of WSIs, slide-level foundation models have also been developed [16, 74]. These operate on larger image regions to capture slide-level context, often by building upon embeddings generated by patch-level encoders (many of which are trained via SSL) in a hierarchical fashion [30, 32]. The quality of these patch-level SSL encoders is thus a critical performance gateway for such hierarchical systems.

### 4.2 Diffusion models

Diffusion models [24] have established themselves as the main approach for building large-scale generative foundation models. Significant advances in training paradigms [51, 62, 42], model architectures [59, 55] , guidance techniques [25, 78, 22], and sampling strategies [61, 46] have enabled these models demonstrate exceptional capabilities in synthesizing high-fidelity, diverse images. Latent Diffusion Models (LDMs) [58], specifically, reduce the computational cost by compressing the images with a learned encoder-decoder pair [76]. As also observed by early works on diffusion models [15], accurate conditioning plays a critical role in achieving high-quality image generation.

Examining popular diffusion foundation models in natural images (SD-XL [56], SD-3 [18], PixArt [12, 10]), most utilize the LDM approach with a pre-trained encoder and decoder, and train on extensive datasets of billions of image-text pairs to train a text-conditioned diffusion model. Beyond data augmentation [68, 4, 6], the powerful image priors encoded by such foundational generative models have been effectively utilized for a wide range of dense prediction tasks, including image segmentation [67] and depth estimation [34]. A primary factor contributing to the success of diffusion models in natural imaging is the availability of vast, curated collections of image-text pairs. To illustrate, Stable Diffusion 1.4 [59] was trained on LAION-5B [60], a dataset comprising 5.85 billion such pairs. In comparison, datasets of this scale and nature are not readily available for histopathology.

Despite these pronounced differences, diffusion models have been explored for digital pathology [48, 49, 75, 3]. Most are limited to training models on small-scale datasets, partly due to the lack of annotations for training a conditional diffusion model on large datasets. Notably, learned representation-guided diffusion models (LRDMs) [21] provide an alternative by training the generative model conditioned on an embedding produced by a self-supervised encoder. This approach offers a path to scaling up diffusion models in the histopathology domain.

## 5 Conclusion

We developed PixCell, the first generative foundation model for histopathology images. Trained on PanCan-30M – an extensive dataset derived from 69,184 H&E-stained whole-slide images covering a comprehensive range of cancer types, PixCell demonstrates state-of-the-art image generation quality. It produces high-fidelity and diverse samples that can effectively serve as a replacement for real images in training self-supervised learning models. We explored PixCell’s capability for controllable generation by conditioning on cell segmentation maps and performing synthetic data augmentation for downstream tasks. Finally, we demonstrated PixCell’s adaptability by showcasing its generalization to different staining techniques, which we used to infer IHC staining from H&E images.

We are publicly releasing the PixCell model weights and associated code to foster future research and applications in computational pathology. Our work with PixCell highlights the potential for diffusion-based generative models to serve as true foundational models within the histopathology domain, offering capabilities that extend beyond traditional discriminative approaches. We hope that this contribution will inspire the broader research community to further utilize these models for innovative applications and novel downstream tasks.

## Figures and Tables

**Table 1:** Details of the data sources used in the PanCan-30M dataset. We extract the 1024 × 1024 patches from whole-slide images across various organs from each data source.

Data Source	# WSI	# Patches

TCGA - diagnostic	11,766	9,192,388
TCGA - fresh frozen	18,310	3,698,529
CPTAC	6,124	2,200,109
SBU	5,698	6,689,518
GTex normal slides	25,713	7,896,145
Other public sources	1,573	1,143,288

Total	69,184	30,819,977

**Table 2:** Training details of PixCell. We progressively scale the model training resolution and resources.

Resolution	# Images	Training steps	Batch size	# GPUs	Conditioning

256 × 256	480 Mil	120,000	4096	16	1 × 1536
512 × 512	120 Mil	60,000	1536	24	4 × 1536
1024 × 1024	30 Mil	80,000	384	32	16 × 1536

**Table 3:** Grid search to find the optimal classifier-free guidance scale w based on the FID score.

w	1	1.2	1.4	1.6	1.8	2	3	5

PixCell-256	13.25	12.21	11.16	10.67	10.34	**9.65**	9.81	10.54
PixCell-1024	8.39	**7.92**	8.55	9.14	9.72	10.83	15.2	20.59

**Table 4:** FID scores for 256 × 256 patches generated by different models.

	Vanilla FID ↓	
	
Method	TCGA-BRCA	PanCan-Test

LRDM [21]	6.98	14.16
ZoomLDM [77]	**6.77**	12.04
PixCell-256	8.79	**9.65**

**Table 5:** Embedding similarity between real and synthetic 256 × 256 patches on PanCan-Test.

	Embedding similarity ↑		
	
Method	UNI-v1	Phicon-v2	CONCH-v1

LRDM [21]	0.408	0.562	0.758
ZoomLDM [77]	0.590	0.682	0.825
PixCell-256	**0.699**	**0.827**	**0.888**

**Table 6:** Image quality of 1024 × 1024 patches and generation time on TCGA-BRCA. PixCell-1024 significantly outperforms all previous baselines in sampling time and generation quality.

Method	Time / img (s)	Crop FID ↓	CLIP FID ↓

LRDM [21]	60	15.51	7.43
∞-Brush [38]	30	17.87	3.74
ZoomLDM [77]	28	14.94	1.23
PixCell-1024	**2.5**	**7.92**	**0.68**

**Table 7:** k-NN performance of SSL encoders (DINOv2 ViT-S) trained on 1M real and synthetic patches from the SBU dataset. We use the Thunder framework [37] for evaluation on patch classification datasets and report the balanced accuracy.

Pretraining Dataset	BACH [2]	BreakHis [63]	NCT-CRC [33]	Patch Camelyon [70]	Average

Synthetic SBU-1M	51.6	62.8	90.4	81.5	71.6
Real SBU-1M	50.0	65.1	92.3	79.5	71.7

**Table 8:** Quantitative evaluation on HER2 stain translation.

Method	SSIM ↑	FID ↓	KID ↓	Crop FID ↓

Baseline [40]	**0.1945**	54.28	16.0	33.22
PixCell-1024 (no LoRA)	0.1880	67.68	19.1	41.54
PixCell-1024	0.1892	**52.32**	**13.4**	**20.87**

**Figure 11:**
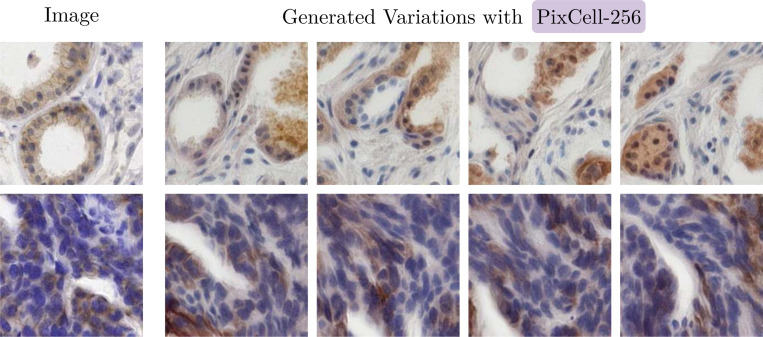
Using an out-of-distribution IHC image as reference, we generate variations using PixCell-256. Although never trained on IHC data, our model can synthesize images from this unseen domain.

**Figure 12:**
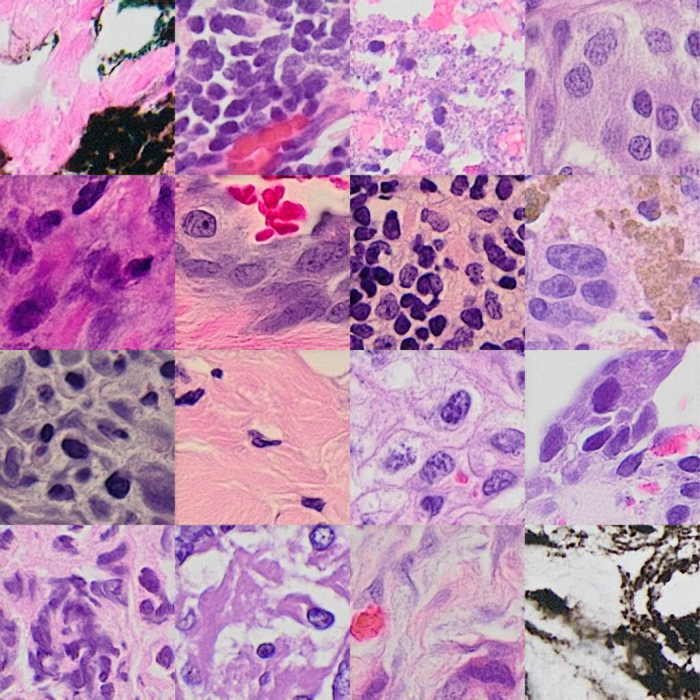
Samples from our Synthetic-SBU-1M dataset. All images in this dataset are generated by providing embeddings from the Real-SBU-1M internal dataset to the PixCell-256 model.

**Figure 13:**
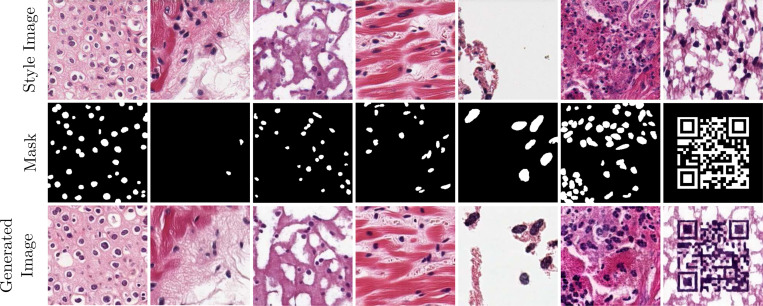
Additional examples of our PixCell-256 cell ControlNet generation.

**Figure 14:**
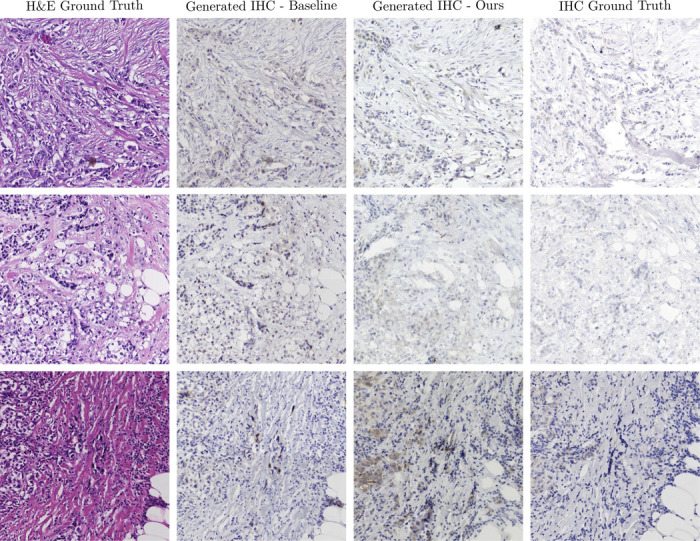
Additional examples of our PixCell-1024 strain translation and the CycleGAN baseline of MIST [40]

**Figure 15:**
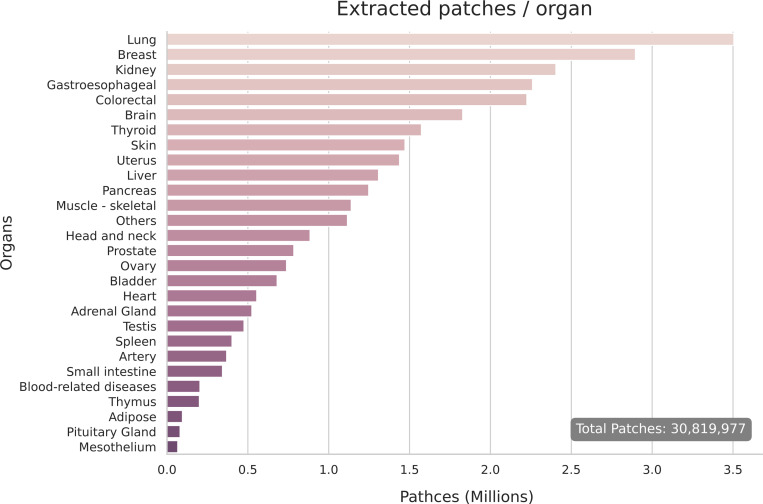
Distribution of organs after patch extraction.

## Footnotes

1 PSNR measured on 1000 random 256 × 256 patches from PanCan-Test.

## A Appendix

### A.1 Additional results:

In this section, we present additional results from our experiments. In Figure 11, we demonstrate the generalization capabilities of PixCell-256 by generating unseen IHC image patches. In Figure 12, we show example images from our Synthetic-SBU-1M dataset. In Figure 13, we provide additional examples of PixCell-256-Cell-ControlNet generation. In Figure 14, we present additional results of our stain translation approach, and the corresponding result from the CycleGAN baseline of MIST [40].

### A.2 Training details

We use an NVIDIA DGX A100-based cluster for our training, image quality evaluation, and self-supervised learning experiments. The DGX A100 node comprises eight NVIDIA A100 GPUs providing 320 GB of memory in total. The node contains two AMD EPYC 7742 CPUs with 256 cores and 1 TB of DDR4 memory. The machine uses an SSD for data storage, which offers up to 25 GB/s in bandwidth. In total, we used 3000 node hours for the paper, including exploratory experiments.

For the controllable generation and stain translation experiments, we used NVIDIA RTX A5000 GPUs with 24GB of memory. Training ControlNets and LoRA-based adapters on our PixCell models can be done on as little as a single A5000 GPU, a compute budget trivial in comparison to the PixCell training requirements. We will be publicly releasing the ControlNet and LoRA training scripts.

### A.3 Dataset details

In Figure 15, we visualize the distribution of organ types in the PanCan-30M dataset after patch extraction. In Table 9, we present the detailed composition of PanCan-30M, including organ types, data sources, dataset splits, and number of WSIs and patches per data source.

**Table 9:** Detailed description of the pretraining dataset used in training the PixCell-256 and PixCell-1024 models.

Organ	Dataset	Split	No of WSIs	No of patches

Adipose	GTEx	Adipose - Subcutaneous	978	57,791
	GTEx	Adipose - Visceral (Omentum)	815	37,763

Adrenal Gland	TCGA FFPE	acc	227	250,571
	TCGA FF	acc	96	11,322
	GTEx	Adrenal Gland	717	263,138

Artery	GTEx	Artery - Aorta	858	274,691
	GTEx	Artery - Coronary	662	33,361
	GTEx	Artery - Tibial	979	61,050

Bladder	GTEx	Bladder	130	61,382
	TCGA FFPE	blca	457	459,281
	TCGA FF	blca	469	79,797
	SBU	Bladder	57	80,421

Blood-related	TCGA FFPE	dlbc	44	26,340
	TCGA FF	dlbc	59	12,427
	CPTAC	AML	93	164,842

Brain	GTEx	Brain - Cerebellum	426	154,674
	GTEx	Brain - Cortex	428	149,441
	CPTAC	GBM	462	99,722
	TCGA FFPE	lgg	844	563,253
	TCGA FF	lgg	728	125,824
	TCGA FFPE	gbm	860	492,950
	TCGA FF	gbm	1193	228,187
	SBU	Brain	15	14,253

Breast	GTEx	Breast - Mammary Tissue	894	144,924
	CPTAC	brca	653	162,981
	TCGA FFPE	brca	1133	735,448
	TCGA FF	brca	1978	312,557
	SBU	Breast	1895	1,540,837

Colorectal	TCGA FFPE	coad	459	267,397
	TCGA FF	coad	983	196,568
	TCGA FFPE	read	165	76,709
	TCGA FF	read	365	72,122
	GTEx	Colon - Sigmoid	800	281,009
	GTEx	Colon - Transverse	937	303,477
	CPTAC	coad	372	126,416
	SBU	Appendix	199	149,278
	SBU	Cecum	61	5,544
	SBU	Colon	354	546,203
	SBU	Rectum	295	200,305

Gastroesophageal	TCGA FFPE	esca	158	114,285
	TCGA FF	esca	238	35,297
	TCGA FFPE	stad	442	305,349
	TCGA FF	stad	755	271,865
	GTEx	Stomach	939	391,985
	GTEx	Esophagus - Gastroesophageal	787	363,692
	GTEx	Esophagus - Mucosa	963	211,581
	GTEx	Esophagus - Muscularis	958	420,428
	SBU	Esophagus	101	19,980
	SBU	Gastroesophageal Junction	2	3
	SBU	Stomach	154	125,812

Head and neck	CPTAC	HNSCC	390	112,431
	TCGA FFPE	hnsc	472	312,557
	TCGA FF	hnsc	791	79,785
	GTEx	Minor Salivary Gland	247	23,017
	SBU	Epiglottis	1	1
	SBU	Gingiva	3	3,510
	SBU	Larynx	171	133,126
	SBU	Lip	44	49,189
	SBU	Maxilla	63	46,149
	SBU	Parotid	53	45,155
	SBU	Submandibular Gland	9	15,571
	SBU	Tongue	10	41,621
	SBU	Tonsil	6	22,393

Heart	GTEx	Heart - Atrial Appendage	614	246,393
	GTEx	Heart - Left Ventricle	761	308,624

Kidney	GTEx	Kidney - Cortex	557	223,111
	GTEx	Kidney - Medulla	49	19,411
	TCGA FFPE	kich	121	104,411
	TCGA FF	kich	205	86,226
	TCGA FFPE	kirc	519	449,337
	TCGA FF	kirc	1654	494,982
	TCGA FFPE	kirp	300	242,237
	TCGA FF	kirp	473	103,243
	CPTAC	CCRCC	884	192,805
	SBU	Kidney	112	489,008

Liver	TCGA FFPE	chol	39	48,090
	TCGA FF	chol	71	13,171
	TCGA FFPE	lihc	379	335,514
	TCGA FF	lihc	491	82,474
	GTEx	Liver	610	272,481
	SBU	Bile Duct	56	1,823
	SBU	Gallbladder	53	49,131
	SBU	Liver	259	505,170

Lung	TCGA FFPE	luad	541	398,649
	TCGA FF	luad	1067	209,038
	TCGA FFPE	lusc	512	380,402
	TCGA FF	lusc	1100	210,390
	GTEx	Lung	860	297,908
	CPTAC	LUAD	969	520,881
	CPTAC	LSCC	1018	488,212
	SBU	Lung	5	630
	others	NLST	1225	999,154

Mesothelium	TCGA FFPE	meso	87	52,567
	TCGA FF	meso	88	14,085
	SBU	Peritoneum	1	1

Muscle - skeletal	CPTAC	SAR	305	95,257
	TCGA FFPE	sarc	600	639,211
	TCGA FF	sarc	290	48,382
	GTEx	Muscle - Skeletal	1001	356,578
	SBU	Stomach	154	125,812

Other Types	TCGA FFPE	pcpg	196	184,298
	TCGA FF	pcpg	189	25,688
	GTEx	Nerve - Tibial	975	95,623
	SBU	Bone	29	51,297
	SBU	Cervix	2	11,402
	SBU	Fallopian Tube	7	320
	SBU	Lymph Node	238	442,527
	SBU	Mediastinum	18	22,158
	SBU	Mesentery	9	1
	SBU	Negative	11	2,303
	SBU	Omentum	10	148
	SBU	Peripheral Nerve	6	1,076
	SBU	Soft Tissue	61	143,760
	SBU	Spinal Cord	5	1,387
	SBU	Unknown	102	106,210
	SBU	Ureter	17	27,609

Ovary	TCGA FFPE	ov	107	102,005
	TCGA FF	ov	1374	372,215
	GTEx	Ovary	255	105,532
	SBU	Ovary	17	15,135
	others	PTRC-HGSOC	348	144,134

Pancreas	TCGA FFPE	paad	209	168,177
	TCGA FF	paad	257	42,614
	GTEx	Pancreas	865	374,094
	CPTAC	PDA	567	98,216
	SBU	Ampulla	142	90,211
	SBU	Pancreas	449	473,837

Pituitary Gland	GTEx	Pituitary	428	81,116

Prostate	GTEx	Prostate	599	262,357
	TCGA FFPE	prad	449	363,268
	TCGA FF	prad	723	139,815
	SBU	Prostate	5	18,765

Skin	TCGA FFPE	uvm	80	46,105
	TCGA FF	uvm	70	8,454
	TCGA FFPE	skcm	475	387,694
	TCGA FF	skcm	475	75,806
	GTEx	Skin - Not Sun Exposed	818	333,848
	GTEx	Skin - Sun Exposed	1001	359,139
	CPTAC	CM	411	138,346
	SBU	Skin	116	122,049

Small intestine	GTEx	Small Intestine - Terminal	798	180,567
	SBU	Duodenum	56	22,758
	SBU	ileum	12	9,905
	SBU	Small Bowel	99	129,820

Spleen	GTEx	Spleen	874	376,920
	SBU	Spleen	5	24,999

Testis	TCGA FFPE	tgct	254	240,765
	TCGA FF	tgct	159	27,182
	GTEx	Testis	592	208,431
	SBU	Stomach	154	125,812

Thyroid	TCGA FFPE	thca	519	398,380
	TCGA FF	thca	639	94,571
	GTEx	Thyroid	902	283,835
	SBU	Thyroid	285	796,642

Uterus	GTEx	Cervix - Ectocervix	38	13,062
	GTEx	Cervix - Endocervix	42	14,578
	GTEx	Fallopian Tube	43	4,979
	GTEx	Uterus	237	106,294
	GTEx	Vagina	276	137,860
	TCGA FFPE	ucs	91	99,858
	TCGA FF	ucs	63	9,524
	TCGA FFPE	cesc	279	175,492
	TCGA FF	cesc	325	49,798
	TCGA FFPE	ucec	566	601,015
	TCGA FF	ucec	805	143,477
	SBU	Uterus	16	82,294
	SBU	Vagina	1	1
